# Challenges and Future Perspectives on Electroencephalogram-Based Biometrics in Person Recognition

**DOI:** 10.3389/fninf.2018.00066

**Published:** 2018-10-09

**Authors:** Hui-Ling Chan, Po-Chih Kuo, Chia-Yi Cheng, Yong-Sheng Chen

**Affiliations:** ^1^Department of Computer Science, National Chiao Tung University, Hsinchu, Taiwan; ^2^Institute of Biomedical Engineering, National Chiao Tung University, Hsinchu, Taiwan; ^3^Center for Emergent Functional Matter Science, National Chiao Tung University, Hsinchu, Taiwan

**Keywords:** electroencephalography (EEG), biometrics, person recognition, person authentication, person identification

## Abstract

The emergence of the digital world has greatly increased the number of accounts and passwords that users must remember. It has also increased the need for secure access to personal information in the cloud. Biometrics is one approach to person recognition, which can be used in identification as well as authentication. Among the various modalities that have been developed, electroencephalography (EEG)-based biometrics features unparalleled universality, distinctiveness and collectability, while minimizing the risk of circumvention. However, commercializing EEG-based person recognition poses a number of challenges. This article reviews the various systems proposed over the past few years with a focus on the shortcomings that have prevented wide-scale implementation, including issues pertaining to temporal stability, psychological and physiological changes, protocol design, equipment and performance evaluation. We also examine several directions for the further development of usable EEG-based recognition systems as well as the niche markets to which they could be applied. It is expected that rapid advancements in EEG instrumentation, on-device processing and machine learning techniques will lead to the emergence of commercialized person recognition systems in the near future.

## Introduction

Biometrics is regarded as a promising alternative to conventional ID cards, keys and passwords in ubiquitous access control systems. This approach provides high commonality, uniqueness, easy acquisition, persistence, portability and resistance to fakery. Biometrics involves quantifying the physical, biological, or behavioral characteristics of individuals, such as fingerprints, iris and retina scans, facial recognition, voice recognition, signatures, palm prints, hand geometry and gait. However, physical traits suffer the risk of violent snatch, whereas explicit behaviors can be observed and imitated. Furthermore, the discriminatory information of fingerprints (the most widely-used biometric trait), has not yet been fully exploited, and its admissibility for trials has been challenged due to the possibility of falsification (Pankanti et al., 2002).

In the late 1990’s, electroencephalography (EEG) was discovered to carry genetically-specific information. The potential of EEG-based biometrics was demonstrated through the successful identification of individuals using features extracted from EEG data acquired during resting states (Poulos et al., 1999a,b,c, 2002). The persistence of individual characteristics in EEG data has yet to be investigated; however, it has the inherent advantage of implicit features, which are difficult to forge. Most efforts into EEG research have focused on understanding how the brain works, the identification of biomarkers, and the construction of brain-computer interfaces (BCIs). BCI systems bypass the motor pathway to establish direct communication between machines and humans. Discriminative features are extracted from EEG signals and classified into various mental states, which are then associated with corresponding control commands for machines. Thus, extracted features should be universally shared within the user population to accommodate inter-subject variation. Conversely, EEG-based identification systems aim to differentiate among individuals performing the same requested task. In this case, any discrepancies in the extracted features tend to facilitate the recognition of individual identities.

**Figure 1:**
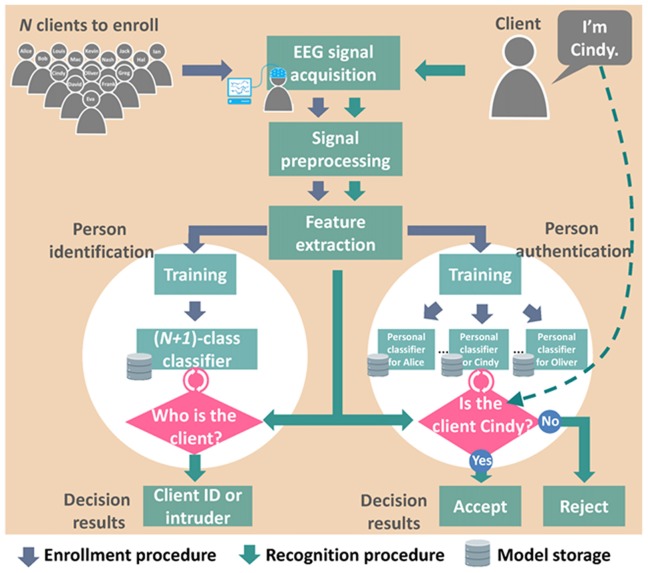
Architecture of person identification and person authentication systems based on electroencephalography (EEG) biometrics.

EEG-based biometrics are applicable to person recognition applications, including identification (Palaniappan and Ravi, 2006; Miyamoto et al., 2008; Hu, 2009; Nakanishi et al., 2009; Fraschini et al., 2015; Rahman and Gavrilova, 2016) and authentication (Marcel and Millán Jdel, 2007; Zúquete et al., 2010; Ashby et al., 2011; Klonovs et al., 2013; Mohanchandra et al., 2013; Yeom et al., 2013; Soni et al., 2016; Nguyen et al., 2017; Thomas and Vinod, 2017) systems. Personal identification systems predict the identity of a user from among all enrolled clients, whereas authentication systems validate the identity claimed by a user. Despite differing purposes, both systems make decisions based on the EEG features of the user and all clients in the database and therefore share the following four components: a database, an EEG acquisition system, a signal preprocessing system, and a feature extraction system (Figure 1). Most identification systems train classifiers to find the best candidate using 1-to-N matching (where N denotes the number of enrolled clients; Poulos et al., 1999c), whereas most authentication systems construct personal classifiers for each client in the database and verify each claim based on the corresponding classifier using 1-to-1 matching (Karthikeyan and Sabarigiri, 2011). To reduce vulnerability to fraud, identification systems can classify intruders as a predefined class using 1-to-(N + 1) matching, and authentication systems can set a threshold in each personal classifier for the rejection of invalid claims.

The performance of EEG-based person recognition systems relies on the design of signal acquisition protocols (Hema et al., 2008), feature extraction methods (Lin et al., 2011) and classification techniques (Palaniappan and Ravi, 2006). Previous reviews (Campisi and Rocca, 2014; Del Pozo-Banos et al., 2014; Abo-Zahhad et al., 2015) have reported on state-of-the-art methods and issues pertaining to the design of each component used in EEG-based biometric systems. As outlined in Yang and Deravi (2017), the overall usability of existing EEG-based biometric systems has been increasing steadily since 2010. However, these systems are still far from commercialization. Our goal in this study was to review recent EEG-based biometric systems and address the gaps impeding their implementation as practical identification or authentication systems. We also outline future perspectives for the deployment of EEG traits in biometric systems. The remainder of this article is divided into four major sections. Section “Advantages of EEG-Based Person Recognition” describes the advantages and potential of EEG-based biometrics in person recognition. Challenges associated with the development of an applicable EEG-based person recognition system are discussed in section “Challenges of EEG-Based Person Recognition.” Section “Future Directions” presents suggestions and research directions for future work. Conclusions are drawn in the section “Conclusion.”

## Advantages of EEG-Based Person Recognition

The use of biometrics to identify individuals or authenticate a personal identity requires measurable physical or behavioral characteristics capable of satisfying the following considerations: universality, distinctiveness, collectability, circumvention, permanence, acceptability and performance (Zhang, 2000; Abo-Zahhad et al., 2015). As a potential biometric trait, EEG signals not only satisfy the first four considerations, they are superior to other biometric traits in this regard. The acceptability, non-invasiveness and privacy afforded by this type of data acquisition makes EEG-based biometric systems a strong contender for widespread public acceptance, as outlined in the next subsection. However, the permanence and performance of EEG-based biometrics is still under research, as discussed in the next section.

### Universality

Many biometric traits are unsuited to people with specific diseases or disabilities. Speech-impaired users are unable to use voice-based biometric systems, and those without hands are unable to use fingerprint-based systems. In contrast, all human brains are composed of neurons that produce electrical activity, which can be read as EEG signals. These signals are accessible in individuals of any age and any mental state, including a vegetative state (Kulkarni et al., 2007) or coma (Young, 2000).

### Distinctiveness

Previous studies have demonstrated the high recognition accuracy of EEG biometrics within a small group of people. La Rocca et al. (2014) proposed a person identification system using functional connectivity during eye-closed (EC) and eye-open (EO) conditions as features. They achieved 100% recognition accuracy among 108 subjects. The CEREBRE system proposed by Ruiz-Blondet et al. (2016) integrates EEG features contributed by various functional brain systems. They achieved 100% recognition among 50 subjects. Thomas and Vinod (2017) proposed a person authentication system using the power spectrum density (PSD) of resting-state EEG signals as features. They achieved an equal error rate (EER) of just 0.008 among 70 subjects. However, the distinctiveness of EEG characteristics among a large population has yet to be investigated. Research into this topic would require the collection of data from many subjects as well as collaboration between research groups. This, in turn, requires the means by which to share data efficiently, as discussed in section “Data Sharing.”

### Collectability

EEG is widely used in clinical applications due to its portability and affordability, compared to other neuroimaging technologies. Unfortunately, EEG signals are recorded using electrodes attached to the scalp using conductive paste to reduce skin impedance, which can be time-consuming and inconvenient for routine biometric procedures. Recent advances in materials and electronic technologies have led to the development of numerous dry EEG electrodes. These gel-free technologies expand the applicability of EEG beyond the clinic. Researchers have also demonstrated that the performance of dry electrodes is on par with that of conventional wet electrodes.

### Circumvention

Many conventional biometrics are easily forged or collected without one’s consent. Fingerprints can be stolen from a cup that the user has held and voices and faces can be recorded in secret. However, to the best of our knowledge, no technique has been developed to enable the reproduction of brainwaves. Furthermore, recording the brainwaves of subjects requires their agreement and cooperation. Even if an individual were to be coerced into allowing recording of their brainwaves, their subsequent negative emotions would produce brainwaves that differ from the templates in the database, leading to rejection by the system.

Some existing biometrics, such as fingerprints and iris and facial scans, are non-cancelable and have a limited number of features. If a system using these biometrics was compromised, then the users may run out of biometric features, leaving them unable to provide alternative data for person recognition. Conventional systems using IDs and passwords can be implemented with password expiration policies, password constraints and user IDs. However, differences among systems can make it difficult for users to remember their personal information. When users are forced to write down their passwords, the system is rendered vulnerable to attack. In EEG-based biometric systems, the EEG acquisition protocols can be adjusted in order to take advantage of a variety of biometric features. It also prevents the leakage of information pertaining to personal identification and authentication by users.

### Friendly Privacy

Facial images and fingerprints can often be obtained without the consent of subjects, but this is not the case for brainwaves. Tracing an individual based on facial images, signatures, or voices is elementary, but EEG data is difficult to obtain. Even if an EEG data storage system were compromised, it would be difficult to find the true identity of a person based on the features of his EEG data. This greatly enhances the security of enrolled clients.

## Challenges of EEG-Based Person Recognition

As described in the previous section, EEG-based biometrics are a feasible alternative to existing methods of person recognition, providing a high level of security. Several studies have reported that EEG-based identification and authentication systems are capable of high recognition accuracy and high permanence; however, many of these systems were evaluated under unrealistic conditions. In the following section, we outline a number of challenges that must be dealt with to improve usability and practicability.

### Operations

The most daunting challenges in the implementation of EEG-based biometrics systems lie in their operation. Most previous EEG experiments have been conducted in laboratories where subjects participated only once or a few times. In reality, however, most biometrics systems are used in a very different way. The system is often accessed repeatedly every day over a period of several years. Thus, attracting users depends on system reliability (discussed in the following sections), as well as ease of use with regard to devices and protocols. Furthermore, the universality of the system requires that protocols be easy and efficient to be performed and applicable to all users including those with physical disabilities. A usable system must be free from devices that require extensive or complicated setup. Finally, it must be possible for users to operate the system on their own, that is, without the need for an operator.

### Stability of System Performance

**Figure 2:**
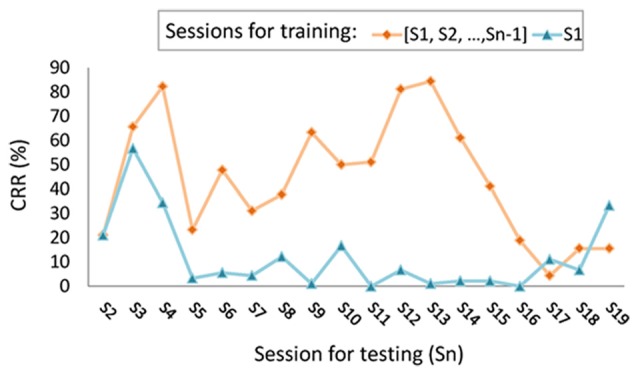
Longitudinal variations in correct recognition rate (CRR) using two-stage person identification system with/without incremental learning, depicted by orange diamonds and blue triangles, respectively (figure depicted according to the results of self-paced finger movement experiment presented in our previous work, Cheng, 2013).

A usable person authentication or identification system must have the ability to recognize enrolled clients, even when they return days, weeks, or years later. In the study of Marcel and Millán Jdel (2007), the half total error rate (HTER) of EEG-based authentication system increased from 7.1 to 36.2 within just 3 days. This trend was also observed in the study of Hu et al. (2011), where the performance of the identification system was evaluated over various time spans. The true positive rate (TAR) after a 1-day span was 94.60%; however, this dropped to 83.64% after a span of 1 week and to 78.20% after 6 months. In our previous work on person recognition using finger-lifting EEG data (Cheng, 2013), the classifier trained using data acquired in the previous days showed fluctuations in the classification recognition rate (CRR) when tested using newly acquired data, as shown in Figure 2. These studies demonstrate the problem of template aging (Yang and Deravi, 2017), in which the performance of an EEG-based biometric system degrades over time. Few studies have examined the stability of their systems over long spans of time. Table 1 lists previous person identification systems alongside a longitudinal examination of their performance over spans exceeding 100 days.

**Table 1:** Longitudinal studies for electroencephalography (EEG)-based person identification.

Reference	Number of users	Number of channels	Protocol	Features	Classifier	Period (day)	Accuracy		Index
							1 st sess.	2 nd sess.	
Näpflin et al. (2007)	20	3	EC	PSD, peak height, peak frequency	General linear model	450	−	88.0	CRR
Näpflin et al. (2008)	20	3	Working memory	PSD, peak height, peak frequency	General linear model	450	−	88.0	CRR
Hu et al. (2011)	11	1	EC	AR, max power, peak frequency, sum power	k-nearest neighbors	182	94.6	78.2	TAR
Kostílek and Št’astný (2012)	9	53	EC, movement	AR	Mahalanobis distance	365	97.4	77.6	CRR
Cheng (2013)	14	12	Movement	PSD	Support vector machine	365	87.7	42.9	CRR
Armstrong et al. (2015)	45	1	Acronym	N400	Cross-correlation	156	98.0	93.0	IA
Ruiz-Blondet et al. (2017)	20	26	CEREBRE	ERP	Cross-correlation	282	100.0	100.0	CRR

*EC, eye-closed resting state; PSD, power spectrum density; AR, autoregressive model; CRR, correct recognition rate; TAR, true positive rate; IA, identification accuracy; sess., session*.

### Robustness to Psychological and Physiological Changes

Achieving stable high recognition accuracy requires that the EEG traits selected as biometric features have high stability and repeatability over time and under a variety of conditions. This is complicated by constant changes in brainwaves over time. EEG-based biometric features are extracted from event-related potentials (ERPs; Marcel and Millán Jdel, 2007; Cheng, 2013; Armstrong et al., 2015) and resting-state recordings (Hu et al., 2011; Kostílek and Št’astný, 2012; Maiorana et al., 2016). The accumulated evidence indicates that ERP and resting-state EEG signals reflect the influences of intrinsic or extrinsic factors, such as aging (Dushanova and Christov, 2014; Sleimen-Malkoun et al., 2015; Kropotov et al., 2016), pain (Schulz et al., 2012), diseases (Jeong, 2004; Han et al., 2013), mental states (Al-Shargie et al., 2016) and emotional states (Lee and Hsieh, 2014; Iacoviello et al., 2015). A good biometric system should be robust to the above conditions; otherwise, enrolled clients may be rejected by the system when they are under stress, pain, or other emotions. To the best of our knowledge, no previous study has addressed the stability and robustness of EEG-based biometric features under relevant-to-life conditions.

### Equipment

The equipment used in EEG-based systems plays an important role when moving out of the laboratory into real-world applications. Recent devices based on dry electrodes provide a good solution to EEG-based biometrics systems; however, the quality of the data is somewhat limited. Differences in the characteristics of data acquired from wet and dry electrodes (Guger et al., 2012) means that the extraction methods devised for one system are not necessarily applicable to the other. Furthermore, the wireless transfer of data (such as Bluetooth) can greatly undermine security. To achieve high precision recognition, the required computing power of modern machine learning techniques is generally high. To build an on-device system, the EEG-based person recognition can only be implemented in high-end consumer products, such as smartphone and laptop, which may ensure privacy but leads to a high cost. Considering the development of artificial-intelligence chips, the cost of on-device processing continues decreasing. The recognition system is also possible to work with cloud computing, on condition that the security and privacy issues of data transfer are carefully considered.

### User Databases

Databases impose four basic challenges: (1) number of users: as the number of subjects increases, it becomes increasingly difficult for the system to accurately classify users. Previous studies have used between 3 and 120 participants. Applying these methods to larger populations may require the sharing of databases. (2) Variations among users: a system for general use should be accessible to a wide variety of subjects; therefore, factors, such as age and sex, must be taken into account by researchers when recruiting users. (3) Variations at the individual level: most previous studies collected data from individual subjects once only. However, as mentioned previously, the brain changes over time and one’s mental state can have a tremendous influence on brain activity. These changes could result in differences between the data used for training and that used for testing. A usable system must be able to capture features that remain stable over long durations. This can only be verified by data collected from the same user several times over an extended period of time. (4) Consideration of intruders: incoming clients can be categorized as legal clients, imposters and intruders (Riera et al., 2007). Legal clients should be correctly identified by the system. Intruders are individuals without any data stored in the database and should be rejected by the system. Imposters are individuals posing as legal clients with data in the user database but they claim their identities as other legal clients.

### Protocol Design

Several protocols have been used for EEG systems, such as non-task (resting state; Poulos et al., 1999c, 2002; Paranjape et al., 2001; Mohammadi et al., 2006; Qinglin et al., 2010; Su et al., 2010; Hu et al., 2011), motor and motor imagery tasks (Shiliang, 2008; Hu, 2009; e.g., hands movement), tasks with sensory inputs (e.g., visual/auditory stimuli; Palaniappan and Mandic, 2005; Palaniappan and Ravi, 2006; Malinka et al., 2011) and cognitive tasks (Marcel and Millán Jdel, 2007; Touyama and Hirose, 2008; Das et al., 2009). Cognitive tasks include those involving counting, problem-solving, mental rotations, letter composing/reading/spelling, memory retrieval and object recognition. Previous studies have found that the brain activation patterns and networks in the resting-state brain are reproducible (Kuntzelman and Miskovic, 2017), such that this would make a suitable protocol for biometric systems. Generally, protocols involving tasks are more reproducible than those without tasks, such as resting-state brain signals. Furthermore, protocols that involve simple tasks based on sensory inputs are more reproducible than those with complex tasks requiring cognitive processing. However, there is a tradeoff between reproducibility and distinctiveness. Protocols capable of evoking brain activity patterns with personal characteristics are regarded as particularly suitable. Brain activity patterns generated during cognitive tasks are distinctive between individuals, which makes them useful in biometric systems; however, these tasks also tend to be time-consuming. Another consideration for protocol design is the repetition effect or neural adaptation, which is the physiological phenomenon induced by repeated stimuli. One way to prevent the neural adaptation is to track the changes of the responses or to use relatively stable characteristics of the signals. For example, although the peak latency and amplitude of visual response may be reduced, the duration of neural activity related to visual processing is not affected by repeated stimuli (Noguchi et al., 2004). Another way to prevent adaptation is to change the contents of stimuli. For example, previous study has adopted emotional faces and found the adaptation can be reduced, comparing to the use of neutral faces in face identification tasks (Gerlicher et al., 2014).

### Performance Evaluation

Once the systems have been developed, researchers report on the accuracy of their systems compared to existing systems. Unfortunately, the lack of standardized evaluation methods by which to obtain an objective evaluation of performance makes such comparisons difficult. Furthermore, most studies report only on recognition accuracy and omit many essential details.

In the following text, we address some of the issues pertaining to evaluation: (1) testing data should be independent or nearly independent from the training data. Most previous studies have applied leave-one-out or k-fold cross-validation for evaluation. When using cross-validation methods, the means by which data is partitioned can have a significant effect on the results. Conducting realistic simulations requires separating training and testing data according to the time at which the data was recorded, rather than simply imposing a random partition. A more rigorous approach would be to formulate one training set and then apply the model to an independent testing set for evaluation. The means by which the model parameters were determined should also be clearly reported. A number of studies have reported results only after tuning the parameters for cross-validation, thereby imposing bias. Testing should be performed using parameters determined exclusively by training data. (2) Additional indices of performance should be reported. Accuracy can be represented as TAR; however, the false positive rate (FAR) is particularly important when the system is used for authentication. When TAR and FAR are both taken into account, receiver operating characteristic (ROC) curves can be plotted to assist in determining the system threshold. (3) It is important that researchers report the duration of the EEG data used or the information transfer rate (ITR). The signal-to-noise ratio (SNR) can be increased by averaging a larger number of epochs due to the whitening property of the noise. However, a larger number of epochs implies that more time is required for data acquisition, which is an important consideration in biometric systems. (4) In studies with a small sample size, the accuracy of results should be tested using statistical methods that take the size of the sample as well as variations between subjects into account. For example, with less testing data, the chance level for two-class classification is not precisely 50% (Kuntzelman and Miskovic, 2017). It is for this reason that conventional parametric methods, such as the *t*-test, are applied against the null hypothesis. Determination of whether accuracy is significantly high requires calculation of confidence intervals at a significant level that depends on the number of trials. Moreover, non-parametric methods such as permutation tests enable better statistical inference than do parametric methods (Maris and Oostenveld, 2007), due to the fact that they are not based on assumptions pertaining to the distribution of the data.

### Uniqueness of EEG Traits Among Twins and Relatives

Previous studies have demonstrated that some EEG features are highly heritable, including band power, alpha frequency, alpha-peak latency, P300 amplitude and P300 latency (van Beijsterveldt and van Baal, 2002; Smit et al., 2006). Moreover, oscillations in the occipital areas have higher heritability than do those in frontal areas (Zietsch et al., 2007). Heritable EEG traits are less susceptible to developmental plasticity and individual experience (Smit et al., 2006). Due to their high stability, heritable EEG traits are potential candidates for biometric features; however, these genetically determined traits tend to present a high degree of similarity between twins or among relatives (Stassen et al., 1987), thereby undermining the distinctiveness of the system. Thus, user databases should include twins and relatives in order to evaluate distinctiveness during performance evaluations.

## Future Directions

In the previous two sections, we discuss the advantages of EEG-based person recognition and describe some of the issues that must be dealt with in the development of a usable system. In this section we provide suggestions with regard to future research to overcome these issues.

### User-Friendly Design

A user-friendly EEG biometric system can really only be achieved using a small number of dry electrodes. Electrodes with conductive gel are acceptable only in cases where there are only a few channels. The forehead is a suitable location for the placement of electrodes, as this facilitates attachment and removal without the hindrance of hair. The number and the placement of electrodes can be further determined by the tasks. More EEG channels can be deployed when using dry electrodes, as in the headset produced by Cognionics or EMOTIV. This kind of headset provides wearable form factors which can be setup in a fast and easy way. Ear-EEG is another comfortable approach by which the EEG can be acquired from the electrodes placed on an earpiece (Mikkelsen et al., 2015). However, the qualities of signals measured by these novel modalities need further improvement before they can be practically applied. A practical EEG biometric system requires that the protocols be: (1) accessible; (2) time-efficient; (3) reproducible; and (4) practice-free (that is, user does not need to practice operation of the system). Time-efficiency is essential and is evaluated by the time for both data acquisition and analysis. Due to the recent advancement of computational capacity for data analysis, the bottleneck usually occurs in the data acquisition procedure in which the EEG signals are recorded for a period of time to increase the SNR. However, our previous work has shown that the accuracy can achieve 85.5% using single trial data (about 1 s per trial) and even 94.7% using the average of two trials (Cheng, 2013). Reproducibility refers to the situation in which brain signals corresponding to particular tasks remain invariant (within the same subject) across several trials and/or over time. Practice-free implementation is based on brain activity features that can be generated naturally (that is, without the need for practice procedures, such as P300, visual-evoked potential and auditory-evoked potential). Such tasks can induce stable EEG signals and are less affected by the environment. Moreover, environment-independent components can be separated from the measurements by using component extraction methods such as independent component analysis and principal component analysis. On the other hand, tasks that require mental practice, such as motor imagery, are inappropriate to be used as EEG-based biometrics, although the tasks are able to evoke brain activity patterns containing personal characteristics.

### Longitudinal Studies and Incremental Learning

The problem of template aging can be overcome by ensuring the completeness of the representation for each enrolled client prior to the training of classifiers. This means that for each individual, EEG data obtained under various conditions and at different times should be included in the training data sets. In particular, some substances may affect brain activity, such as medicine (Banoczi, 2005; Blume, 2006), tobacco (Tcheslavski, 2008), nicotine (Knott and Fisher, 2007), caffeine (Meng et al., 2017), or alcohol (Kähkönen et al., 2003). In Marcel and Millán Jdel (2007), the authentication model was updated using newly acquired EEG recordings based on the incremental learning method. These measures were shown to mediate the drop in performance over a span of 3 days. In recent developments of biometrics in smartphone authentication, the models used for face or fingerprint recognition are adjusted during every login procedure. In a previous study, we applied incremental learning to an EEG-based person identification system (Cheng, 2013). Longitudinal variation was examined by having one of the 23 participants repeatedly (19 times within 2 years) conduct a finger movement experiment. Each session of EEG data, S_*n*_ (*n* = 2, 3, …, 19), was used to test the following: (1) the identification classifier trained using the first session (S_1_); and (2) the identification classifier trained using all of the data acquired in the previous sessions, [S_1_, S_2_, …, S_*n*-1_]. In 16 of the 18 testing sessions, the second identification model achieved better CRR than did the first model, as shown in Figure 2. These results demonstrate the feasibility of incremental learning and indicate the importance of acquiring complete training data in order to maintain performance. We therefore strongly recommend the use of longitudinal EEG acquisition and performance evaluation during training steps in order to improve the temporal persistence of EEG-based biometric systems.

### Modeling Psychological and Physiological Changes

**Figure 3:**
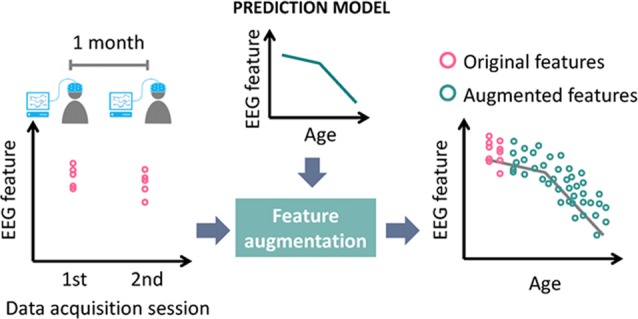
Diagram of feature augmentation using prediction model (age taken as example of factor affecting EEG features).

EEG measurements can be affected by psychological and physiological factors. Increased delta power has been observed in mice with sleep deprivation (Curie et al., 2013). Medication may cause the power increase of beta and theta bands (Blume, 2006). Negative and positive emotional states induce different patterns of functional connectivity (Chan et al., 2012, 2015b; Lee and Hsieh, 2014). Patients suffering from Parkinson’s disease exhibit slower EEG in theta band than that of the normal controls (Soikkeli et al., 1991). Thus, developing a stable and effective EEG-based biometric system requires an understanding of the factors affecting EEG, as well as a means of selecting EEG features with high stability and distinctiveness. The resulting model can be used to perform data augmentation through the artificial enlargement of the dataset and improved classifier training (Krizhevsky et al., 2012). This model also makes it possible to predict changes in features, thereby enabling the biometric system to maintain high recognition accuracy over time. Take aging as an example, where the power of auditory event-related low frequency oscillations decreases over time (Dushanova and Christov, 2014). If the oscillation power were used as a feature, then a classifier trained using features augmented in accordance with the aging model might recognize clients even as they aged. The concept of feature augmentation using a prediction model is illustrated in Figure 3. The accumulation of abundant knowledge concerning the influence of various factors makes it possible for researchers to build a model capable of making accurate predictions of EEG features under varying conditions. The use of a prediction model in conjunction with feature augmentation could greatly reduce the time required to obtain training data.

### Multitasking and Multimodal Biometrics

Conventional biometric systems rely on a single biometric signature for person recognition; however, biometrics with multiple tasks or multiple modalities can improve the accuracy of person recognition and also increase the difficulty of forging biometric data.

A number of multiple-task methods have been proposed to improve system reliability. The CEREBRE system demonstrated 100% identification accuracy (IA) by integrating EEG features from multiple tasks (Ruiz-Blondet et al., 2016, 2017). In Palaniappan (2008), the authors combined five tasks to train classifiers: resting, math activity, geometric rotation, letter composing and visual counting. Unlike training classifiers in which data is collected from all tasks at once, multitask learning constructs a decision model by considering the main task separately from the extra tasks (Shiliang, 2008). Acquiring EEG data from a variety of tasks can be time-consuming because the tasks must be performed sequentially; however, statistical methods can be used to find the optimal sequence of stimuli and shorten experimental time (Dale, 1999). One accessible design scenario for multiple tasks in a biometric system involves a series of tasks in a single paradigm rather than having users perform several tasks one by one. This can be regarded as a system capable of extracting different features during different time periods. For example, a single paradigm with three components could be formulated as follows: visual-evoked potentials (P100) followed by face-evoked potential (N170), and then face recognition (after N250). For paradigms with more than two types of stimuli, a statistical experimental design can help to shorten the time required to conduct the experiment, while still acquiring a sufficient number of data points.

Multimodal biometrics is another way to improve recognition accuracy (Shekhar et al., 2014; Kumar and Kumar, 2016) without unduly extending the time required for data acquisition. For example, a multimodal biometric system can record EC resting-state activity of a client and perform the facial recognition at the same time. During the execution of finger-lifting tasks, the event-related potentials and fingerprints can be recorded simultaneously.

Fusion with EEG-based biometrics is one potential solution to the two major issues posed by conventional biometrics: spoofing-attack detection and liveness detection (Akhtar et al., 2015). EEG-based biometric systems can easily perform liveness detection because only living individuals have brainwaves. Determining whether the provision of data is voluntary is also fairly straightforward because people present different EEG characteristics in stressful situations. EEG biometrics also has lower circumvention, compared to other biometrics.

**Figure 4:**
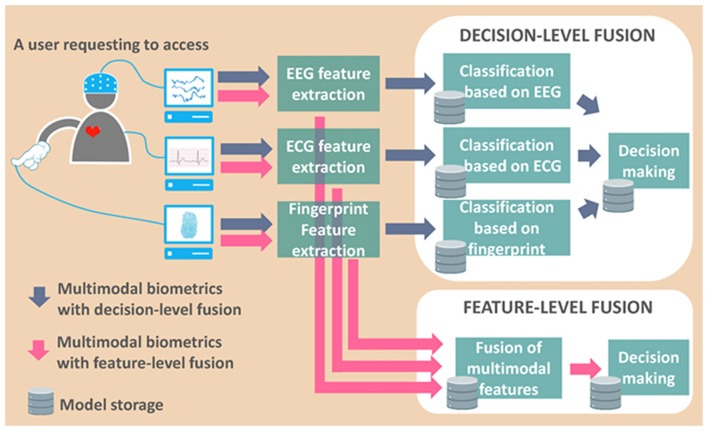
Diagram of data processing flow in multimodal biometric system (enrollment procedure not shown).

We suggest combining EEG biometric features with conventional biometrics to compensate for weaknesses on both sides. Facial and fingerprint IDs are good candidates for conventional biometrics due to their effectiveness, stability, popularity and low cost. Researchers should also be aware that the performance of multimodal biometric systems relies on the selection of fusion parameters, such as the weight of each biometric feature, and the decision rules used to compile a score from each of the biometric classifiers (Kumar and Kumar, 2016). Figure 4 presents an example of fusing EEG, ECG and fingerprint features to illustrate the flow of multimodal data processing when a client requests access. Deep learning (DL) is another approach to the fusing of data from EEG and other biometric modalities. This approach has been applied to computing representations of multimodal biometrics for spoofing detection (Menotti et al., 2015). This technique has recently attracted attention due to its high performance. Further details on DL are presented in the next subsection.

### Machine Learning for EEG-Based Biometrics

**Figure 5:**
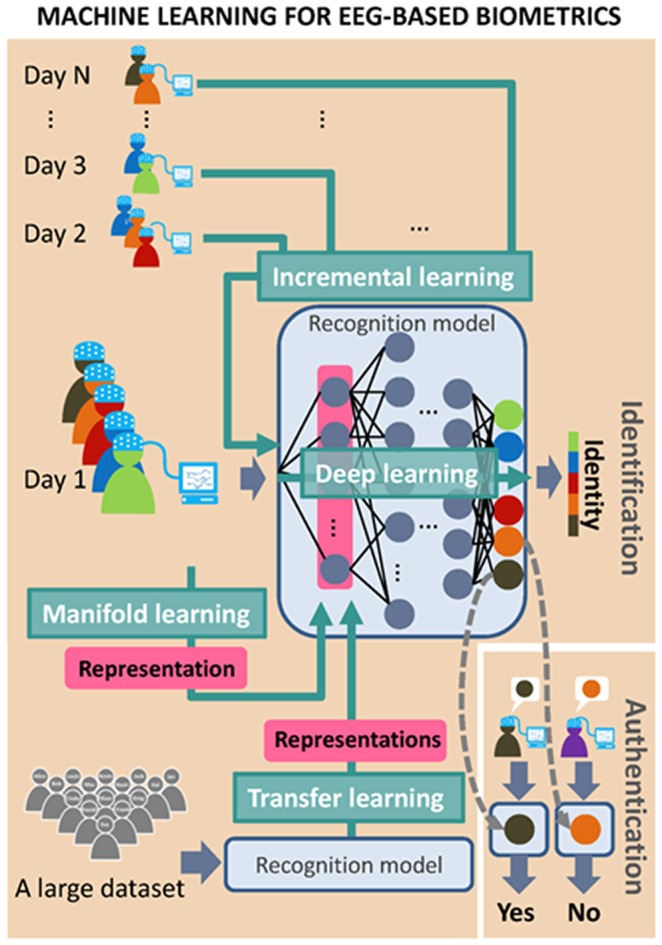
Diagram of four machine learning techniques: incremental learning, Deep learning (DL), transfer learning and manifold learning, which can be applied in EEG-based biometrics.

Machine learning methods can help to improve the system performance by adapting the model to the data. Figure 5 illustrates the concepts of using four machine learning methods for EEG-based person recognition. Incremental learning can be used for modeling longitudinal data of EEG signals (as mentioned in the previous section), in order to obtain a realistic spatiotemporal representation of EEG signals. DL is a popular machine learning method, which can learn a set of features and construct a deep neural network for a specific purpose, such as object recognition. Some recent studies have applied DL techniques to the classification of EEG signals from patients as well as normal subjects (Zhao and He, 2015). Some BCI studies also used DL methods to classify brain signals into different categories corresponding to different instructions (Schirrmeister et al., 2017). When using DL to improve an EEG-based biometric system, the main problem is determining the means by which to classify EEG signals of different users. Once the trained neural network is able to identify users, it can also be used to extract user-specific features from EEG data. These types of user-specific feature can be applied to authentication systems for the calculation of similarity between the test and the true user. DL might also be a feasible approach to modal combination, in determining the fusion parameters capable of achieving the highest accuracy.

Transfer leaning (TL) is another important machine learning technique applicable to EEG-based biometric systems. The idea behind TL is to learn a representative structure from a large database and then share this structure to adjust models lacking training data. In BCI systems, the purpose of TL is to find common structures across subjects for use in refining the model for each subject (Zanini et al., 2018). This strategy can also be used in biometrics, wherein a system based on a model capable of classifying a large number of users is transferred to another system with fewer users in order to improve classification accuracy. TL can also be used for the extraction of features, which are then transferred from the identification system to an authentication system.

Manifold learning is another method by which to formulate a good representation of data in a low-dimensional space. Manifold learning has recently been used in the analysis of MEG data based on the assumption that the brain transforms high-dimensional sensory inputs into a low-dimensional representation (Kuo et al., 2014). A manifold is constructed by preserving the local distance between data (that is, spatiotemporal brain activity) and enlarging the distance between data that is further apart in the original high-dimensional space. This makes it possible to keep together (close) brain activity belonging to a particular individual after the transformation, while pulling apart instances of brain activity belonging to other individuals. However, DL, TL and manifold learning require a large amount of training data to overcome problems imposed by the high dimensionality associated with the sharing of data.

### Data Sharing

Several databases are used for fingerprinting, such as NIST SD27, WVU database and the IIITD database, which can be used to help developers test their products. The major limitation of current studies on EEG-based biometrics is an inadequate number of subjects. One emerging trend in the field of neuroimaging is the sharing of data to improve reproducibility, reduce the cost of research, and maximize the contribution of scientific resources (Poldrack and Gorgolewski, 2014). There are more than 8,000 shared structured magnetic resonance images (MRIs) and more than 1,000 functional MRIs online (e.g., HCP and OpenfMRI); however, EEG databases contain data exclusive to that particular study. For example, some datasets contain only the data collected from patients or individuals with disorders that are inapplicable to biometric applications. Once a wide range of EEG data becomes available, researchers will be able to develop and test their systems without the need to collect large amounts of data.

A generalized dataset has recently made a significant contribution to the development of DL. We suggest that a shared EEG database with large variability among the subjects (for example, encompassing different races and ages) would be useful in the development of systems aimed at universality. However, two important issues must be mentioned: (1) confidential information, such as names, ages, and genders of subjects as well as other behavioral data must be protected or encrypted. Before experiments, subjects must be informed in advance and give formal consent. (2) The format of the data can vary considerably due to differences in equipment type, channel layout, sampling rates, impedance and bandpass filters. Minor factors that are generally overlooked (for examples, experiment location or conditions of subjects) could also make a big difference, and should therefore be recorded. Not just raw data, but even data with different analytical levels (e.g., after preprocessing or after frequency component extraction) could be shared. Raw data requires a great deal of space for storage, but it has tremendous potential for reuse. Nonetheless, an authorized standard must be formulated in order to establish future EEG biometric systems, and only EEG data that fits the standard would be included in the database. This would help to ensure the successful transfer of data or features from one system to another. When developing a new system, a standardized EEG database could help developers to obtain meaningful evaluations of their work. We strongly recommend that researchers in EEG biometrics (previous as well as ongoing) share their data within an accepted standard, such that all subsequent studies would be able to train their models and validate their results with greater ease and effect.

### Two-Stage System to Minimize FAR While Keeping TAR

Instances of false acceptance permit invalid access and information leakage. Thus, systems requiring high security depend on an access control system that minimizes FAR. However, many of the methods used to prevent false acceptance may also decrease TAR or increase the false rejection rate (FRR). A biometric system with low TAR or high FRR necessitates the inclusion of alternative methods such as passwords.

**Figure 6:**
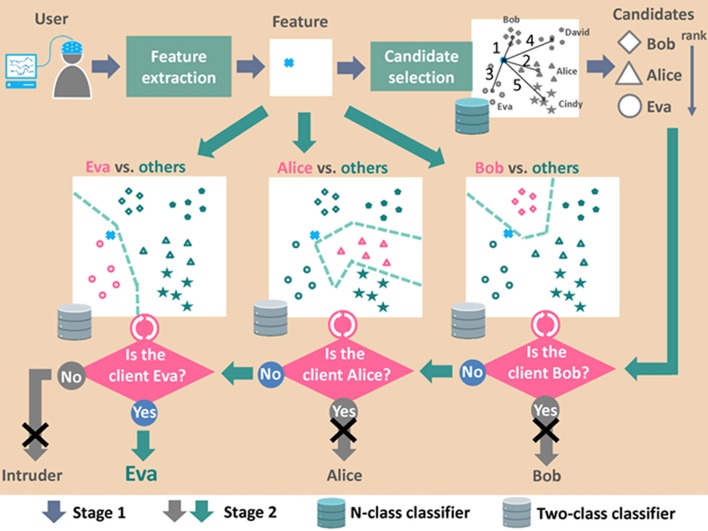
Diagram of data processing flow in two-stage person identification system. Note that the enrollment procedure is not shown in this figure. The figure was depicted based on the concept of data processing flow presented in our previous work (Cheng, 2013).

FAR can be minimized while maintaining high TAR by dividing the process of recognition into two stages: (1) one stage is meant to reduce FAR; and (2) the other stage is meant to increase TAR. In Palaniappan (2008), researchers proposed a two-stage person authentication system: (1) potential impostors are identified in the first stage to reduce FAR; then (2) measures are taken to verify their status as impostors in order to reduce FRR. That approach was shown to achieve zero FAR and zero FRR in four of the five subjects. In our previous work (Cheng, 2013), we propose a two-stage person identification system in which one-against-rest classifiers are applied to multiple candidates individually and sequentially until the identity of a single candidate is verified, as shown in Figure 6. Our two-stage identification system achieved TAR of 97.1% and FAR of 0.6%. The same system without one-against-rest verification gave TAR of 98.1% and FAR of 1.9%. Clearly, the two-stage framework is applicable to the development of applications requiring high security.

### Exploring Additional EEG Features

In the following text, we present three methods for the extraction of additional features for use in EEG-based biometrics. Non-linearity features the complexity of signals, and previous studies have shown that brain activity contains a non-linear structure. Thus, we can deduce that conventional linear analysis would be insufficient for such tasks (Stam, 2005; Gao et al., 2015; Kuo et al., 2017). Neural complexity can be regarded as irregularity of activity in the brain, which is related to brain functions and information processing (McDonough and Nashiro, 2014). Therefore, differences in the complexity of data embedded in EEG can be used to differentiate among entities. Entropy is one measure by which to assess the complexity of the brain, and it has also been used in person authentication (Mu et al., 2016). Calculating entropy at various temporal scales makes it possible to formulate a complete description of the non-linearity in EEG signals (Gao et al., 2015). We suggest using both linear and non-linear features of EEG signals with different temporal scales as a means of increasing accuracy.

The second approach is the use of connectivity in the human brain, including functional and effective connectivity (Friston, 2011), to assess the interactions between various regions of the brain. Functional connectivity measures the statistical dependence between activity in spatially segregated regions using linear indices, such as correlation (Chan et al., 2015b), and non-linear indices, such as phase locking values (Tass et al., 1998) and phase-amplitude coupling (Tort et al., 2010; Chan et al., 2015a). Effective connectivity quantifies the causal effects among various neural systems using approaches such as dynamic causal modeling (Kiebel et al., 2009) and Granger causal modeling (David et al., 2006). Moreover, changes in brain connectivity have been associated with the effects of aging (Ferreira et al., 2016), cognitive decline (Onoda et al., 2012), disease (de Hemptinne et al., 2015) and emotional states (Lee and Hsieh, 2014). Thus, brain connectivity has considerable potential for use in modeling psychological and physiological changes.

Non-cortical components, such as the signals evoked by eye-movement are the third type of potential feature. Eye-movement components in EEG are conventionally treated as artifacts and removed prior to analysis. In one study, subjects were asked to perform specific movements such as eye blinking and mouth moving, whereupon the artifacts evoked in the EEG signals were used for person authentication (Pham et al., 2014). Thus, it is possible to adopt artifacts in EEG as additional features for use in conjunction with the artifact-free EEG signals to improve system performance.

### Niche Markets

Compared to the sensors used to acquire fingerprints, voice samples, or facial features, current EEG devices are large and expensive and suffer from a low SNR, which has hampered the applicability of EEG-based person recognition. The medical and consumer electronics industries are pushing the development of EEG devices for consumers, which are smaller, more portable, and cheaper than the EEG devices currently used in clinics. Despite hardware limitations, EEG-based biometric systems have a number of advantages (particularly with regard to security) over other types of biometric system, as discussed in section “Advantages of EEG-Based Person Recognition.” At present, EEG-based person recognition systems are best suited to applications that require high security and have low requirement on portability. Ideally, the system would be able to tolerate slow responses and the administrators would value security sufficiently to overlook the high cost. Examples include the securing of top-secret documents in governments or companies, accessing control of bank coffers, or of the use of authorization codes for weapons of mass destruction. The rapid growth of machine learning research leads to the improvement of recognition performance. Moreover, the development of artificial-intelligence chips points out the possibility of on-device processing for EEG-based biometrics. Along with the improvements of the portability and sensitivity of electrophysiological instruments, EEG-based person recognition stands a good chance to be applied to security administration of consumer electronics, including smartphones, smart door locks and laptops.

The tolerance of recognition errors depends on application scenarios. The threshold should be strictly set for the applications with high demand of security, such as home door locker and bank coffers. In these situations, the environments are relatively stable, allowing strict threshold and low tolerance of recognition errors in EEG biometric systems. Moreover, EEG-based emotion recognition (Alarcao and Fonseca, 2018) should also be applied to block coerced users, who usually show strong fear and stress. On the other hand, the authentication for smartphone and laptop are usually used in various environments. The acceptance threshold should be loosened to avoid frequent rejection caused by deviation of EEG signals induced by ambient interferences as well as distraction and emotion states of users.

## Conclusion

Explosive growth in digital technology has resulted in a plethora of applications requiring IDs and passwords, and the amount of personal information stored in the cloud is increasing rapidly. This has necessitated the development of access control methods capable of reducing the burden on users while simultaneously fortifying security. A review of the literature confirms that EEG-based biometrics cannot be lost by users and are difficult to steal or forge. Thus, EEG has considerable potential for use in person recognition systems. However, processing efficiency, recognition accuracy and user-friendly designs must evolve before commercial EEG-based person recognition systems are viable. An EEG-based biometric system requires highly sensitive and portable EEG dry electrodes, a simple task design for data acquisition, signal features with high stability and high distinctiveness and an adaptive and accurate machine learning classifier. This article outlines a number of methods by which to overcome the disadvantages of existing EEG-based person recognition systems, and thereby promote the emergence of EEG-based person identification or authentication for security control in the near future.

## Author Contributions

H-LC coordinated the manuscript writing process. H-LC and P-CK reviewed related articles and drafted the manuscript. C-YC provided the data based on her master thesis study. Y-SC reviewed and revised the manuscript.

## Conflict of Interest Statement

The authors declare that the research was conducted in the absence of any commercial or financial relationships that could be construed as a potential conflict of interest.

## References

[B1] Abo-Zahhad M. Ahmed S. M. Abbas S. N. (2015). State-of-the-art methods and future perspectives for personal recognition based on electroencephalogram signals. IET Biom. 4, 179–190. 10.1049/iet-bmt.2014.0040

[B2] Akhtar Z. Micheloni C. Foresti G. L. (2015). Biometric liveness detection: challenges and research opportunities. IEEE Secur. Priv. 13, 63–72. 10.1109/msp.2015.116

[B3] Alarcao S. M. Fonseca M. J. (2018). Emotions recognition using EEG signals: a survey. IEEE Trans. Affect. Comput. [Epub ahead of print]. 99 10.1109/TAFFC.2017.2714671

[B4] Al-Shargie F. Kiguchi M. Badruddin N. Dass S. C. Hani A. F. M. Tang T. B. (2016). Mental stress assessment using simultaneous measurement of EEG and fNIRS. Biomed. Opt. Express 7, 3882–3898. 10.1364/BOE.7.003882 27867700 PMC5102531

[B5] Armstrong B. C. Ruiz-Blondet M. V. Khalifian N. Kurtz K. J. Jin Z. Laszlo S. (2015). Brainprint: assessing the uniqueness, collectability and permanence of a novel method for ERP biometrics. Neurocomputing 166, 59–67. 10.1016/j.neucom.2015.04.025

[B6] Ashby C. Bhatia A. Tenore F. Vogelstein J. (2011). “Low-cost electroencephalogram (EEG) based authentication,” in 5th International IEEE/EMBS Conference on Neural Engineering (NER), Cancun, Mexico.

[B7] Banoczi W. (2005). How some drugs affect the electroencephalogram (EEG). Am. J. Electroneurodiagnostic. Technol. 45, 118–129. 10.1080/1086508X.2005.11079518 15989074

[B8] Blume W. T. (2006). Drug effects on EEG. J. Clin. Neurophysiol. 23, 306–311. 10.1097/01.wnp.0000229137.94384.fa 16885705

[B9] Campisi P. Rocca D. L. (2014). Brain waves for automatic biometric-based user recognition. IEEE Trans. Inf. Forensics Secur. 9, 782–800. 10.1109/tifs.2014.2308640

[B11] Chan H.-L. Chen Y.-S. Chen L.-F. (2012). Selection of independent components based on cortical mapping of electromagnetic activity. J. Neural Eng. 9:056006. 10.1088/1741-2560/9/5/056006 22878589

[B12] Chan H.-L. Chen Y.-S. Chen L.-F. Baillet S. (2015a). “Beamformer-based imaging of phase-amplitude coupling using electromagnetic brain activity,” in 37th Annual International Conference of the IEEE Engineering in Medicine and Biology Society (EMBC), Milan, Italy, 7558–7561. 10.1109/EMBC.2015.7320141 26738041

[B10] Chan H.-L. Chen L.-F. Chen I.-T. Chen Y.-S. (2015b). Beamformer-based spatiotemporal imaging of linearly-related source components using electromagnetic neural signals. Neuroimage 114, 1–17. 10.1016/j.neuroimage.2015.03.038 25804642

[B13] Cheng C.-Y. (2013). EEG-Based Person Identification System and Its Longitudinal Adaptation. Master in Computer Science, National Chiao Tung University, Hsinchu, Taiwan.

[B14] Curie T. Mongrain V. Dorsaz S. Mang G. M. Emmenegger Y. Franken P. (2013). Homeostatic and circadian contribution to EEG and molecular state variables of sleep regulation. Sleep 36, 311–323. 10.5665/sleep.2440 23450268 PMC3571738

[B15] Dale A. M. (1999). Optimal experimental design for event-related fMRI. Hum. Brain Mapp. 8, 109–114. 10.1002/(sici)1097-0193(1999)8:2/3<109::aid-hbm7>3.0.co;2-w 10524601 PMC6873302

[B16] Das K. Zhang S. Giesbrecht B. Eckstein M. P. (2009). “Using rapid visually evoked EEG activity for person identification,” in 31st Annual International Conference of the IEEE Engineering in Medicine and Biology Society (EMBC), Minneapolis, MN. 10.1109/IEMBS.2009.5334858 19964968

[B17] David O. Kiebel S. J. Harrison L. M. Mattout J. Kilner J. M. Friston K. J. (2006). Dynamic causal modeling of evoked responses in EEG and MEG. Neuroimage 30, 1255–1272. 10.1016/j.neuroimage.2005.10.045 16473023

[B18] de Hemptinne C. Swann N. C. Ostrem J. L. Ryapolova-Webb E. S. San Luciano M. Galifianakis N. B.. (2015). Therapeutic deep brain stimulation reduces cortical phase-amplitude coupling in Parkinson’s disease. Nat. Neurosci. 18, 779–786. 10.1038/nn.3997 25867121 PMC4414895

[B19] Del Pozo-Banos M. Alonso J. B. Ticay-Rivas J. R. Travieso C. M. (2014). Electroencephalogram subject identification: a review. Expert Syst. Appl. 41, 6537–6554. 10.1016/j.eswa.2014.05.013

[B20] Dushanova J. Christov M. (2014). The effect of aging on EEG brain oscillations related to sensory and sensorimotor functions. Adv. Med. Sci. 59, 61–67. 10.1016/j.advms.2013.08.002 24797977

[B21] Ferreira L. K. Regina A. C. Kovacevic N. Martin Mda G. Santos P. P. Carneiro Cde G.. (2016). Aging effects on whole-brain functional connectivity in adults free of cognitive and psychiatric disorders. Cereb. Cortex 26, 3851–3865. 10.1093/cercor/bhv190 26315689

[B22] Fraschini M. Hillebrand A. Demuru M. Didaci L. Marcialis G. L. (2015). An EEG-based biometric system using eigenvector centrality in resting state brain networks. IEEE Signal Process. Lett. 22, 666–670. 10.1109/lsp.2014.2367091

[B23] Friston K. J. (2011). Functional and effective connectivity: a review. Brain Connect. 1, 13–36. 10.1089/brain.2011.0008 22432952

[B24] Gao J. Hu J. Liu F. Cao Y. (2015). Multiscale entropy analysis of biological signals: a fundamental bi-scaling law. Front. Comput. Neurosci. 9:64. 10.3389/fncom.2015.00064 26082711 PMC4451367

[B25] Gerlicher A. M. V. van Loon A. M. Scholte H. S. Lamme V. A. F. van der Leij A. R. (2014). Emotional facial expressions reduce neural adaptation to face identity. Soc. Cogn. Affect. Neurosci. 9, 610–614. 10.1093/scan/nst022 23512931 PMC4014095

[B26] Guger C. Krausz G. Allison B. Z. Edlinger G. (2012). Comparison of dry and gel based electrodes for P300 brain-computer interfaces. Front. Neurosci. 6:60. 10.3389/fnins.2012.00060 22586362 PMC3345570

[B27] Han C.-X. Wang J. Yi G.-S. Che Y.-Q. (2013). Investigation of EEG abnormalities in the early stage of Parkinson’s disease. Cogn. Neurodyn. 7, 351–359. 10.1007/s11571-013-9247-z 24427211 PMC3713203

[B28] Hema C. R. Paulraj M. P. Kaur H. (2008). “Brain signatures: a modality for biometric authentication,” in International Conference on Electronic Design (ICED), Penang, Malaysia.

[B30] Hu J.-F. (2009). “New biometric approach based on motor imagery EEG signals,” in International Conference on Future Biomedical Information Engineering, Sanya, China.

[B29] Hu B. Liu Q. Zhao Q. Qi Y. Peng H. (2011). “A real-time electroencephalogram (EEG) based individual identification interface for mobile security in ubiquitous environment,” in IEEE Asia-Pacific Services Computing Conference (APSCC), Jeju Island, South Korea.

[B31] Iacoviello D. Petracca A. Spezialetti M. Placidi G. (2015). A real-time classification algorithm for EEG-based BCI driven by self-induced emotions. Comput. Methods Programs Biomed. 122, 293–303. 10.1016/j.cmpb.2015.08.011 26358282

[B32] Jeong J. (2004). EEG dynamics in patients with Alzheimer’s disease. Clin. Neurophysiol. 115, 1490–1505. 10.1016/j.clinph.2004.01.001 15203050

[B33] Kähkönen S. Wilenius J. Nikulin V. V. Ollikainen M. Ilmoniemi R. J. (2003). Alcohol reduces prefrontal cortical excitability in humans: a combined TMS and EEG study. Neuropsychopharmacology 28, 747–754. 10.1038/sj.npp.1300099 12655321

[B34] Karthikeyan D. T. Sabarigiri B. (2011). Enhancement of multi-modal biometric authentication based on iris and brain neuro image coding. Int. J. Biom. Bioinform. 5, 249–256.

[B35] Kiebel S. J. Garrido M. I. Moran R. Chen C. C. Friston K. J. (2009). Dynamic causal modeling for EEG and MEG. Hum. Brain Mapp. 30, 1866–1876. 10.1002/hbm.20775 19360734 PMC6870752

[B36] Klonovs J. Petersen C. K. Olesen H. Hammershoj A. (2013). ID proof on the go: development of a mobile EEG-based biometric authentication system. IEEE Veh. Tech. Mag. 8, 81–89. 10.1109/mvt.2012.2234056

[B37] Knott V. J. Fisher D. J. (2007). Naltrexone alteration of the nicotine-induced EEG and mood activation response in tobacco-deprived cigarette smokers. Exp. Clin. Psychopharmacol. 15, 368–381. 10.1037/1064-1297.15.4.368 17696684

[B38] Kostílek M. Št’astný J. (2012). “EEG biometric identification: repeatability and influence of movement-related EEG,” in International Conference on Applied Electronics, Pilsen, Czech Republic.

[B39] Krizhevsky A. Sutskever I. Hinton G. E. (2012). “Imagenet classification with deep convolutional neural networks,” in Proceedings of the 26th Annual Conference on Neural Information Processing Systems (NIPS), (Lake Tahoe, NV), 1097–1105.

[B40] Kropotov J. Ponomarev V. Tereshchenko E. P. Müller A. Jäncke L. (2016). Effect of aging on ERP components of cognitive control. Front. Aging Neurosci. 8:69. 10.3389/fnagi.2016.00069 27092074 PMC4821853

[B41] Kulkarni V. P. Lin K. Benbadis S. R. (2007). EEG findings in the persistent vegetative state. J. Clin. Neurophysiol. 24, 433–437. 10.1097/WNP.0b013e31815c2810 18090523

[B42] Kumar A. Kumar A. (2016). Adaptive management of multimodal biometrics fusion using ant colony optimization. Inf. Fusion 32, 49–63. 10.1016/j.inffus.2015.09.002

[B43] Kuntzelman K. Miskovic V. (2017). Reliability of graph metrics derived from resting-state human EEG. Psychophysiology 54, 51–61. 10.1111/psyp.12600 28000256

[B45] Kuo P.-C. Chen Y.-T. Chen Y.-S. Chen L.-F. (2017). Decoding the perception of endogenous pain from resting-state MEG. Neuroimage 144, 1–11. 10.1016/j.neuroimage.2016.09.040 27746387

[B44] Kuo P.-C. Chen Y.-S. Chen L.-F. Hsieh J.-C. (2014). Decoding and encoding of visual patterns using magnetoencephalographic data represented in manifolds. Neuroimage 102, 435–450. 10.1016/j.neuroimage.2014.07.046 25072391

[B46] La Rocca D. Campisi P. Vegso B. Cserti P. Kozmann G. Babiloni F.. (2014). Human brain distinctiveness based on EEG spectral coherence connectivity. IEEE Trans. Biomed. Eng. 61, 2406–2412. 10.1109/TBME.2014.2317881 24759981

[B47] Lee Y.-Y. Hsieh S. (2014). Classifying different emotional states by means of EEG-based functional connectivity patterns. PLoS One 9:e95415. 10.1371/journal.pone.0095415 24743695 PMC3990628

[B48] Lin J. P. Chen Y. S. Chen L. F. (2011). “Person identification using electroencephalographic signals evoked by visual stimuli,” in Neural Information Processing, ICONIP 2011. Lecture Notes in Computer Science, eds Lu B. L. Zhang L. Kwok J. (Berlin, Heidelberg: Springer), 684–691. 10.1007/978-3-642-24955-6_81

[B49] Maiorana E. Rocca D. L. Campisi P. (2016). On the permanence of EEG signals for biometric recognition. IEEE Trans. Inform. Forensics Secur. 11, 163–175. 10.1109/tifs.2015.2481870

[B50] Malinka K. Hanacek P. Trzos M. (2011). “Evaluation of biometric authentication based on visual evoked potentials,” in International Carnahan Conference on Security Technology (ICCST), Barcelona, Spain.

[B51] Marcel S. Millán Jdel R. (2007). Person authentication using brainwaves (EEG) and maximum a posteriori model adaptation. IEEE Transactions on Pattern Analysis and Machine Intelligence 29, 743–748. 10.1109/tpami.2007.1012 17299229

[B52] Maris E. Oostenveld R. (2007). Nonparametric statistical testing of EEG- and MEG-data. J. Neurosci. Methods 164, 177–190. 10.1016/j.jneumeth.2007.03.024 17517438

[B53] McDonough I. M. Nashiro K. (2014). Network complexity as a measure of information processing across resting-state networks: evidence from the human connectome project. Front. Hum. Neurosci. 8:409. 10.3389/fnhum.2014.00409 24959130 PMC4051265

[B54] Meng J. Mundahl J. H. Streitz T. D. Maile K. Gulachek N. S. He J.. (2017). Effects of soft drinks on resting state EEG and brain-computer interface performance. IEEE Access 5, 18756–18764. 10.1109/access.2017.2751069 29423352 PMC5798644

[B55] Menotti D. Chiachia G. Pinto A. Schwartz W. R. Pedrini H. Falcao A. X. (2015). Deep representations for iris, face, and fingerprint spoofing detection. IEEE Trans. Inform. Forensics Secur. 10, 864–879. 10.1109/tifs.2015.2398817

[B56] Mikkelsen K. B. Kappel S. L. Mandic D. P. Kidmose P. (2015). EEG recorded from the ear: characterizing the ear-EEG method. Front. Neurosci. 9:438. 10.3389/fnins.2015.00438 26635514 PMC4649040

[B57] Miyamoto C. Baba S. Nakanishi I. (2008). “Biometric person authentication using new spectral features of electroencephalogram (EEG),” in International Symposium on Intelligent Signal Processing and Communications Systems (ISPACS), Bangkok.

[B58] Mohammadi G. Shoushtari P. Ardekani B. M. Shamsollahi M. B. (2006). “Person identification by using AR model for EEG signals,” in International Conference on Computer Science, Prague.

[B59] Mohanchandra K. Lingaraju G. Kambli P. Krishnamurthy V. (2013). Using brain waves as new biometric feature for authenticating a computer user in real-time. Int. J. Biom. Bioinform. 7, 49–57.

[B60] Mu Z. Hu J. Min J. (2016). EEG-based person authentication using a fuzzy entropy-related approach with two electrodes. Entropy 18:432 10.3390/e18120432

[B61] Nakanishi I. Baba S. Miyamoto C. (2009). “EEG based biometric authentication using new spectral features,” in International Symposium on Intelligent Signal Processing and Communication Systems (ISPACS), Kanazawa, Japan.

[B62] Näpflin M. Wildi M. Sarnthein J. (2007). Test-retest reliability of resting EEG spectra validates a statistical signature of persons. Clin. Neurophysiol. 118, 2519–2524. 10.1016/j.clinph.2007.07.022 17892969

[B63] Näpflin M. Wildi M. Sarnthein J. (2008). Test-retest reliability of EEG spectra during a working memory task. Neuroimage 43, 687–693. 10.1016/j.neuroimage.2008.08.028 18817882

[B64] Nguyen B. Nguyen D. Ma W. Tran D. (2017). “Investigating the possibility of applying EEG lossy compression to EEG-based user authentication,” in International Joint Conference on Neural Networks,Anchorage, AK.

[B65] Noguchi Y. Inui K. Kakigi R. (2004). Temporal dynamics of neural adaptation effect in the human visual ventral stream. J. Neurosci. 24, 6283–6290. 10.1523/jneurosci.0655-04.2004 15254083 PMC6729535

[B66] Onoda K. Ishihara M. Yamaguchi S. (2012). Decreased functional connectivity by aging is associated with cognitive decline. J. Cogn. Neurosci. 24, 2186–2198. 10.1162/jocn_a_00269 22784277

[B67] Palaniappan R. (2008). Two-stage biometric authentication method using thought activity brain waves. Int. J. Neural Syst. 18, 59–66. 10.1142/s0129065708001373 18344223

[B68] Palaniappan R. Mandic D. (2005). “Energy of brain potentials evoked during visual stimulus: a new biometric?” in 15th International Conference on Artificial Neural Networks (ICANN), Warsaw.

[B69] Palaniappan R. Ravi K. V. R. (2006). Improving visual evoked potential feature classification for person recognition using PCA and normalization. Pattern Recognit. Lett. 27, 726–733. 10.1016/j.patrec.2005.10.020

[B70] Pankanti S. Prabhakar S. Jain A. K. (2002). On the individuality of fingerprints. IEEE Trans. Pattern Anal. Mach. Intell. 24, 1010–1025. 10.1109/tpami.2002.1023799

[B71] Paranjape R. B. Mahovsky J. Benedicenti L. Koles Z. (2001). “The electroencephalogram as a biometric,” in Canadian Conference on Electrical and Computer Engineering, Toronto, ON.

[B72] Pham T. Ma W. Tran D. Nguyen P. Phung D. (2014). “EEG-based user authentication using artifacts,” in International Joint Conference SOCO’14-CISIS’14-ICEUTE’14: Bilbao, Spain, June 25th–27th, 2014, Proceedings, eds de la Puerta J. G. Ferreira I. G. Bringas P. G. Klett F. Abraham A. de Carvalho A. C. P. L. F. (Cham: Springer International Publishing), 343–353.

[B73] Poldrack R. A. Gorgolewski K. J. (2014). Making big data open: data sharing in neuroimaging. Nat. Neurosci. 17, 1510–1517. 10.1038/nn.3818 25349916

[B75] Poulos M. Rangoussi M. Alexandris N. (1999a). “Neural network based person identification using EEG features,” in IEEE International Conference on Acoustics, Speech and Signal Processing, Phoenix, AZ.

[B76] Poulos M. Rangoussi M. Chrissikopoulos V. Evangelou A. (1999b). “Parametric person identification from the EEG using computational geometry,” in 6th IEEE International Conference on Electronics, Circuits and Systems, Pafos, Cyprus.

[B77] Poulos M. Rangoussi M. Chrissikopoulos V. Evangelou A. (1999c). “Person identification based on parametric processing of the EEG,” in 6th IEEE International Conference on Electronics, Circuits and Systems, Pafos, Cyprus.

[B74] Poulos M. Rangoussi M. Alexandris N. Evangelou A. (2002). Person identification from the EEG using nonlinear signal classification. Methods Inf. Med. 41, 64–75. 10.1055/s-0038-1634316 11933767

[B78] Qinglin Z. Hong P. Bin H. LanLan L. YanBing Q. QuanYing L. (2010). “Towards an efficient and accurate EEG data analysis in EEG-based individual identification,” in 7th International Conference on Ubiquitous Intelligence and Computing (UIC), Xi’an, China.

[B79] Rahman M. W. Gavrilova M. (2016). “Overt mental stimuli of brain signal for person identification,” in International Conference on Cyberworlds (CW), Chongqing, China.

[B80] Riera A. Soria-Frisch A. Caparrini M. Grau C. Ruffini G. (2007). Unobtrusive biometric system based on electroencephalogram analysis. EURASIP J. Adv. Signal Process. 2008:143728 10.1155/2008/143728

[B81] Ruiz-Blondet M. V. Jin Z. Laszlo S. (2016). CEREBRE: a novel method for very high accuracy event-related potential biometric identification. IEEE Trans. Inform. Forensics Secur. 11, 1618–1629. 10.1109/tifs.2016.2543524

[B82] Ruiz-Blondet M. V. Jin Z. Laszlo S. (2017). Permanence of the CEREBRE brain biometric protocol. Pattern Recognit. Lett. 95, 37–43. 10.1016/j.patrec.2017.05.031

[B83] Schirrmeister R. T. Springenberg J. T. Fiederer L. D. J. Glasstetter M. Eggensperger K. Tangermann M.. (2017). Deep learning with convolutional neural networks for EEG decoding and visualization. Hum. Brain Mapp. 38, 5391–5420. 10.1002/hbm.23730 28782865 PMC5655781

[B84] Schulz E. Zherdin A. Tiemann L. Plant C. Ploner M. (2012). Decoding an individual’s sensitivity to pain from the multivariate analysis of EEG data. Cereb. Cortex 22, 1118–1123. 10.1093/cercor/bhr186 21765182

[B85] Shekhar S. Patel V. M. Nasrabadi N. M. Chellappa R. (2014). Joint sparse representation for robust multimodal biometrics recognition. IEEE Trans. Pattern Anal. Mach. Intell. 36, 113–126. 10.1109/TPAMI.2013.109 24231870

[B86] Shiliang S. (2008). “Multitask learning for EEG-based biometrics,” in 19th International Conference on Pattern Recognition (ICPR), Tampa, FL.

[B87] Sleimen-Malkoun R. Perdikis D. Müller V. Blanc J.-L. Huys R. Temprado J.-J.. (2015). Brain dynamics of aging: multiscale variability of EEG signals at rest and during an auditory oddball task. eNeuro 2:ENEURO.0067-14.2015. 10.1523/eneuro.0067-14.2015 26464983 PMC4586928

[B88] Smit C. M. Wright M. J. Hansell N. K. Geffen G. M. Martin N. G. (2006). Genetic variation of individual alpha frequency (IAF) and alpha power in a large adolescent twin sample. Int. J. Psychophysiol. 61, 235–243. 10.1016/j.ijpsycho.2005.10.004 16338015

[B89] Soikkeli R. Partanen J. Soininen H. Paakkonen A. Riekkinen P. Sr. (1991). Slowing of EEG in Parkinson’s disease. Electroencephalogr. Clin. Neurophysiol. 79, 159–165. 10.1016/0013-4694(91)90134-p 1714807

[B90] Soni Y. S. Somani S. B. Shete V. V. (2016). “Biometric user authentication using brain waves,” in International Conference on Inventive Computation Technologies (ICICT), Coimbatore, India.

[B91] Stam C. J. (2005). Nonlinear dynamical analysis of EEG and MEG: review of an emerging field. Clin. Neurophysiol. 116, 2266–2301. 10.1016/j.clinph.2005.06.011 16115797

[B92] Stassen H. H. Bomben G. Propping P. (1987). Genetic aspects of the EEG: an investigation into the within-pair similarity of monozygotic and dizygotic twins with a new method of analysis. Electroencephalogr. Clin. Neurophysiol. 66, 489–501. 10.1016/0013-4694(87)90095-2 2438114

[B93] Su F. Xia L. Cai A. Wu Y. Ma J. (2010). “EEG-based personal identification: from proof-of-concept to a practical system,” in 20th International Conference on Pattern Recognition (ICPR), Istanbul, Turkey.

[B94] Tass P. Rosenblum M. G. Weule J. Kurths J. Pikovsky A. Volkmann J. (1998). Detection of n:m phase locking from noisy data: application to magnetoencephalography. Phys. Rev. Lett. 81, 3291–3294. 10.1103/physrevlett.81.3291

[B95] Tcheslavski G. V. (2008). Effects of tobacco smoking and schizotypal personality on spectral contents of spontaneous EEG. Int. J. Psychophysiol. 70, 88–93. 10.1016/j.ijpsycho.2008.06.004 18620008

[B96] Thomas K. P. Vinod A. P. (2017). EEG-based biometric authentication using gamma band power during rest state. Circuits Syst. Signal Process. 37, 277–289. 10.1007/s00034-017-0551-4

[B97] Tort A. B. Komorowski R. Eichenbaum H. Kopell N. (2010). Measuring phase-amplitude coupling between neuronal oscillations of different frequencies. J. Neurophysiol. 104, 1195–1210. 10.1152/jn.00106.2010 20463205 PMC2941206

[B98] Touyama H. Hirose M. (2008). “Non-target photo images in oddball paradigm improve EEG-based personal identification rates,” in 30th Annual International Conference of the IEEE Engineering in Medicine and Biology Society (EMBC), Vancouver, BC. 10.1109/IEMBS.2008.4650115 19163618

[B99] van Beijsterveldt C. E. M. van Baal G. C. M. (2002). Twin and family studies of the human electroencephalogram: a review and a meta-analysis. Biol. Psychol. 61, 111–138. 10.1016/s0301-0511(02)00055-8 12385672

[B100] Yang S. Deravi F. (2017). On the usability of electroencephalographic signals for biometric recognition: a survey. IEEE Trans. Hum. Mach. Syst. 47, 958–969. 10.1109/thms.2017.2682115

[B101] Yeom S. K. Suk H. I. Lee S. W. (2013). “EEG-based person authentication using face stimuli,” in International Winter Workshop on Brain-Computer Interface (BCI), Gangwo, South Korea.

[B102] Young G. B. (2000). The EEG in coma. J. Clin. Neurophysiol. 17, 473–485. 10.1097/00004691-200009000-00006 11085551

[B103] Zanini P. Congedo M. Jutten C. Said S. Berthoumieu Y. (2018). Transfer learning: a Riemannian geometry framework with applications to brain-computer interfaces. IEEE Trans. Biomed. Eng. 65, 1107–1116. 10.1109/TBME.2017.2742541 28841546

[B104] Zhang D. D. (2000). Automated Biometrics: Technologies and Systems. South Holland, Netherlands: Kluwer Academic Publishers.

[B105] Zhao Y. He L. (2015). “Deep learning in the EEG diagnosis of Alzheimer’s disease,” in Computer Vision-ACCV 2014 Workshops: Singapore, Singapore, November 1-2, 2014, Revised Selected Papers, Part I, eds Jawahar C. V. Shan S. (Cham: Springer International Publishing), 340–353.

[B106] Zietsch B. P. Hansen J. L. Hansell N. K. Geffen G. M. Martin N. G. Wright M. J. (2007). Common and specific genetic influences on EEG power bands delta, theta, alpha and beta. Biol. Psychol. 75, 154–164. 10.1016/j.biopsycho.2007.01.004 17316957

[B107] Zúquete A. Quintela B. Cunha J. P. S. (2010). “Biometric authentication with electroencephalograms: evaluation of its suitability using visual evoked potentials,” in International Joint Conference on Biomedical Engineering Systems and Technologies (BIOSTEC), Valencia, Spain.

